# Short-Season Direct-Seeded Cotton Cultivation Under Once-Only Irrigation Throughout the Growing Season: Investigating the Effects of Planting Density and Nitrogen Application

**DOI:** 10.3390/plants14121864

**Published:** 2025-06-17

**Authors:** Zhangshu Xie, Yeling Qin, Xuefang Xie, Xiaoju Tu, Aiyu Liu, Zhonghua Zhou

**Affiliations:** 1 Cotton Research Institute, Agronomy College, Hunan Agricultural University, Changsha 410128, China; xiaosu201420182022@stu.hunau.edu.cn (Z.X.); 15773126526@163.com (Y.Q.);; 2 Rubber Research Institute, Chinese Academy of Tropical Agricultural Sciences, Haikou 571101, China; 952189.ok@163.com; 3 Yue Lu Shan Laboratory, Changsha 410128, China

**Keywords:** cotton, short-season direct seeding, once-only irrigation, density, nitrogen application rate, yield, physiological activity

## Abstract

To identify optimal strategies for high-yield and high-efficiency cultivation under a “short-season direct-seeded cotton with once-only irrigation” regime, we conducted two-year field experiments (2022 and 2023) using a split-plot factorial design with three planting densities (30,000 (D1), 45,000 (D2), and 60,000 (D3) plants·ha^−1^) and three nitrogen application rates (150 (N1), 180 (N2), and 210 (N3) kg·ha^−1^). Our study systematically examined how these treatment combinations influenced canopy architecture, physiological traits, yield components, and fiber quality. The results showed that increased planting density significantly enhanced plant height, the leaf area index (LAI), and the number of fruiting branches, with the highest density (D3) contributing to a more compact and efficient canopy. Moderate nitrogen input (N2) significantly increased peroxidase (POD) activity, reduced malondialdehyde (MDA) accumulation, delayed functional leaf senescence, and prolonged the canopy’s photosynthetic performance. A significant interaction between planting density and nitrogen application was observed. The D3N2 treatment (high density with moderate nitrogen) consistently achieved the highest fruiting branch count, boll number per plant, and yields of both seed cotton and lint in both years, while maintaining stable fiber quality. This indicates its strong capacity to balance high yield with quality and maintain physiological resilience. By contrast, the D1N1 treatment (low density and low nitrogen) exhibited a loose canopy, premature photosynthetic decline, and the lowest yield. The D3N3 treatment (high density and high nitrogen) promoted vigorous early growth but reduced stress tolerance during later growth stages, leading to yield instability. These findings demonstrate that moderately increasing planting density while maintaining appropriate nitrogen levels can effectively optimize canopy structure, improve stress resilience, and enhance yield under short-season direct-seeded cotton systems with once-only irrigation. This provides both theoretical underpinning and practical guidance for achieving stable and efficient cotton production under such systems.

## 1. Introduction

Cotton (*Gossypium hirsutum* L.) remains the premier natural fiber crop globally and a key dual-purpose source of fiber and edible oil, sustaining employment, industrial value chains, and foreign-exchange earnings across most producing nations [1]. China occupies the dual role of top producer and consumer: in 2023, 2.788 M ha was sown, yielding 5.62 Mt of lint, while domestic demand approached 7.90 Mt, revealing a pronounced supply deficit [2]. Over the past decade, accelerating urbanization has inflated land and labor costs, undermining the viability of smallholder-dominated systems in the Yellow and Yangtze River basins; outputs there have collapsed from 1.58 Mt and 1.27 Mt in 2010 to 0.24 Mt and 0.22 Mt, respectively, in 2023 [3]. Conversely, Xinjiang leveraged large-scale mechanized harvesting and favorable solar thermal conditions to boost production to 5.11 Mt, exceeding 90% of the national total [2]. While this geographic concentration enhances efficiency, it amplifies vulnerability to extreme weather, pest epidemics, and market volatility, posing systemic threats to national supply security. Accordingly, China’s “14th Five-Year” agricultural modernization plan designates protected cotton production zones with Xinjiang as the core and the Yangtze and Yellow River basins as strategic complements, targeting area stabilization, yield gains, and cost reductions [4]. In this context, Hunan Province—long a staple cotton area within the Yangtze basin, possessing synergistic heat–light–water regimes and ample machine-harvestable land—has been earmarked as a critical inland hub for revitalizing production and diluting regional risk [5].

To reconcile land-use competition between cotton and rapeseed while coping with labor scarcity, Hunan Province is accelerating the adoption of a “short-season direct-seeded cotton” regime characterized by early-maturing varieties, slightly deferred sowing, single-pass mechanical seeding, and synchronized machine harvesting. Condensing the crop cycle to 140–150 days permits seamless annual rotation with rapeseed and simultaneously reduces field operations and labor demand [6]. Nevertheless, the constrained developmental window of short-season cotton heightens its reliance on the precise spatiotemporal coordination of water and nutrient supply [7].

Coupled with the Yangtze Basin’s pronounced rainfall seasonality and intricate irrigation–drainage infrastructure, many growers have adopted a “once-only irrigation” strategy, typically applied at the start of the squaring stage, to reduce labor, energy inputs, and costs while coping with water availability constraints [8]. This practice resembles the deficit irrigation systems widely studied in arid and semi-arid cotton zones [9,10] but is uniquely adapted to monsoonal conditions with abundant early-season rainfall and no supplementary irrigation after initial establishment. Recent studies have explored one-time or minimal irrigation approaches in cotton for improving water productivity and optimizing resource use [11,12], yet most have not focused on rainfed monsoon systems combined with direct-seeded, short-duration cotton cultivars.

Within this context, once-only irrigation fundamentally reshapes field water fluxes, soil nitrogen release, and crop development rhythms, demanding precise control of canopy structure and nutrient uptake. Planting density and nitrogen application become pivotal management levers to orchestrate biomass partitioning, boll setting, and fiber maturation [13]. While prior studies have examined the individual or combined effects of planting density and nitrogen rate under conventional irrigation regimes such as drip or stage-wise irrigation [14,15], systematic quantitative assessments under the once-only irrigation regime—particularly within a resource-constrained, short-season direct-seeding system—remain notably scarce.

We hypothesize that an optimal “density–nitrogen” window exists that matches the one-off irrigation regime, enabling maintenance of or even improvement in yield and fiber quality while minimizing N input. The results will fill the theoretical gap concerning density × nitrogen interactions under the “short-season direct-seeding plus once-only irrigation” model; provide an integrated water-saving, N-reducing, and mechanization-compatible cultivation package for the Yangtze River basin and other monsoon regions; and offer empirical evidence to support the diversification of China’s cotton production zones, thus strengthening supply security and industry resilience.

## 2. Materials and Methods

### 2.1. Experimental Materials

The experimental material used in this study was the early-maturing, directly sown cotton cultivar JX0010 developed by the Cotton Research Institute, Hunan Agricultural University, with a growth period of approximately 105 days. Prior to sowing, the seeds were sulfuric-acid-delinted and sun-dried, and seeds of uniform plumpness were selected for planting.

### 2.2. Experimental Site

The field experiments were conducted in 2022 and 2023 in Yanxi Town, Liuyang City, Hunan Province, China (28°18′19″ N, 113°49′26″ E). The region is characterized by a humid subtropical monsoon climate, with mean annual temperatures and precipitation of 18.8 °C and 1128.5 mm in 2022, and 19.2 °C and 1137.6 mm in 2023, respectively.

The experimental soil is classified as an Ultisol under the USDA Soil Taxonomy and as a Ferralic Cambisol according to the World Reference Base (WRB). The soil texture in the 0–20 cm plow layer is silty clay loam, with moderate granular structure and good permeability. The baseline soil fertility properties are summarized in Table 1.

In 2022 and 2023, the topsoil showed moderately acidic pH values of 5.73 and 5.86, respectively. Organic matter contents were 24.94 g/kg and 25.70 g/kg, with total nitrogen (TN) concentrations of 1.49 g/kg and 1.55 g/kg. Alkaline hydrolyzable nitrogen (AN) contents were 152.15 mg/kg and 153.34 mg/kg, effective phosphorus (Olsen-P) contents were 104.76 mg/kg and 126.13 mg/kg, and available potassium (AK) contents were 150 mg/kg and 120 mg/kg, respectively.

Although macro- and micronutrient concentrations are not fully presented, the soil generally displayed moderate-to-high fertility status for N, P, and K. However, due to limitations in initial analysis, micronutrients (Fe, Mn, Zn, Cu), cation exchange capacity (CEC), soil base saturation (BS, %), and the sum of bases (SB) were not measured. These parameters will be considered in future studies.

### 2.3. Experimental Design

Sowing was carried out on 30 May in both 2022 and 2023 using a two-factor experimental design incorporating planting density and nitrogen rate. Planting densities were set at 30,000 plants·ha^−1^ (D1), 45,000 plants·ha^−1^ (D2), and 60,000 plants·ha^−1^ (D3), while nitrogen rates were 150 kg·ha^−1^ (N1), 180 kg·ha^−1^ (N2), and 210 kg·ha^−1^ (N3).

The selected planting density levels reflect both the regional agronomic recommendations for short-season cotton under mechanized direct seeding [16] and findings from a preliminary field trial conducted in 2022 at the same site, which indicated yield sensitivity to densities below 30,000 and diminishing returns beyond 60,000 plants·ha^−1^. Similarly, the nitrogen rates were chosen based on the standard fertilization guideline for cotton in Hunan Province (150–210 kg N ha^−1^) [17], aligned with regional best practices, and scaled to allow detection of the impacts of both deficient and excessive N application under the once-only irrigation system.

All nitrogen was band-applied in a single dose at the squaring stage to synchronize nutrient release with peak crop demand. The ridge height and furrow width were 10 cm and 30 cm, respectively. Each plot covered 19.2 m^2^ (3.2 m × 6 m), and all treatments were replicated three times, resulting in 27 plots. A single furrow irrigation (~30 m^3^ water) was applied pre-fertilization, with water reaching ridge tops. Other crop management practices followed the regional specification DB43/T 2379-2022 [17].

### 2.4. Measurement Indicators and Methods

#### 2.4.1. Agronomic Traits

Plant height: At four growth stages (seedling stage, bud stage, flower and boll stage, and boll-opening stage), five contiguous plants in each plot were tagged. Plant height was determined as the vertical distance from the soil surface to the apex of the main stem. The mean of the five plants was used for analysis.

Number of fruiting branches: At the bud, flower and boll, and boll-opening stages, the number of fruiting branches on the main stem was counted on the same five tagged plants per plot, and the average value was calculated.

Leaf area index (LAI): At the seedling, bud, flower and boll, and boll-opening stages, the leaf area index of each plot was measured with an AccuPAR LP-80 canopy analyzer (METER Group, Pullman, WA, USA). Four measurements were taken along an S-shaped transect within each plot and averaged.

#### 2.4.2. Physiological Indicators

At the seedling, bud, flower and boll, and boll-opening stages, the fourth leaf from the apex of the main stem was sampled (at boll-opening after topping, the apical leaf of the main stem was collected). Samples were sealed in zip-lock bags, immediately frozen in liquid nitrogen, and transported to the laboratory. Peroxidase (POD) activity and malondialdehyde (MDA) content were determined using commercial kits (Solarbio, Beijing, China) in accordance with the manufacturer’s instructions. Each parameter was measured three times, and the mean value was calculated.

#### 2.4.3. Yield Traits and Yields

Number of bolls per plant: During the boll-opening stage, ten adjacent plants were sampled in each plot, and the count of intact bolls per plant—excluding aborted or rotten bolls—was recorded. The average served as the plot value.

Boll weight: Fifty fully opened bolls were harvested from the mid-to-lower canopy at the boll-opening stage, sun-dried, and weighed for each plot. The average mass was calculated.

Lint percentage: Seed cotton was ginned with a saw gin (model 110A, Dongguang Xinxing Cotton Machinery Factory, Cangzhou, China). Lint percentage was calculated as lint mass divided by seed cotton mass [6].

Seed cotton yield: plant density × bolls plant^−1^ × boll mass/1000 × 0.85 (correction factor).

Lint yield: seed cotton yield × lint percentage.

#### 2.4.4. Fiber Quality

For each plot, 20 g of lint that had been ginned and thoroughly sun-dried was placed in a sealed envelope and sent to the Cotton Quality Supervision, Inspection and Testing Center, Ministry of Agriculture and Rural Affairs (Institute of Cotton Research, Chinese Academy of Agricultural Sciences, Anyang, China). Five HVI parameters—average length of upper half, uniformity index, fiber strength, micronaire value, and elongation rate—were determined according to “Test Method for Physical Properties of Cotton Fiber by HVI”.

### 2.5. Data Processing and Analysis

The data were collated in Microsoft Excel 2010. The variance analyses were conducted with the General Linear Model procedure in SPSS Statistics 25.0 (IBM, Armonk, NY, USA), and Duncan’s multiple range test was used for post hoc mean separation and significance testing.

## 3. Results and Analysis

### 3.1. Changes in Cotton Plant Height Under Different Treatments

Across the two growing seasons, cotton plant height exhibited a consistent upward trajectory throughout development, peaking at the boll-opening stage (Table 2). Plant height responded differentially to stand density, nitrogen application rate, and their interaction.

As for the main effect of density, the highest density treatment (D3) markedly augmented plant height. At the boll-opening stage in 2022, D3 plants were 123.60 cm (10.53%) taller on average than those under the low-density treatment D1 (111.82 cm). The 2023 results mirrored this pattern, suggesting that crowded stands intensify vertical competition and consequently stimulate elongation growth.

With respect to nitrogen supply, the intermediate rate (N2) yielded the maximum plant height. At boll opening in 2022, the plants under N2 treatment reached 123.16 cm—7.30% above the low-N treatment N1 (114.78 cm). The 2023 data corroborated this, as N2 plants (120.16 cm) exceeded N1 (112.53 cm) by 6.78%.

The density–nitrogen interaction revealed that the high-density/medium-nitrogen treatment (D3N2) consistently delivered the tallest plants. At the 2023 boll-opening stage, D3N2 plants attained 127.27 cm, surpassing every other treatment. By contrast, nitrogen effects were negligible under low density: in 2023, the D1N1 and D1N3 plants measured only 104.87 cm and 106.67 cm, respectively, implying that inadequate resource interception at low density limited nitrogen-use efficiency.

Taken together, plant height proved more responsive to stand density than to N supply. A moderate increase in planting density, complemented by an intermediate N rate, markedly stimulated vegetative growth, optimized canopy spatial occupation, and established a favorable basis for later dry matter accumulation and yield.

### 3.2. Changes in the Number of Cotton Fruiting Branches Under Different Treatments

Across the two experimental seasons, the overall temporal pattern was similar, yet the total number of fruiting branches in 2023 was slightly lower than in 2022. Planting density and the nitrogen application rate exerted differential regulatory effects on fruiting branch number (Table 3).

With respect to density effects, the number of fruiting branches at the bud stage in 2023 reached 6.53 under the high-density treatment (D3), which was 58.11% higher than that under the low-density treatment D1 (4.13). At the flower and boll stage, D3 plants produced 11.53 fruiting branches, 33.76% more than D1 (8.62), indicating that high stand density promotes the differentiation and accumulation of fruiting branches by intensifying intraspecific competition.

Regarding nitrogen effects, the medium nitrogen level (N2) demonstrated the highest performance. During the flower and boll stage in 2022, the number of fruiting branches under N2 was 11.69, 24.89% higher than that under low N (N1, 9.36). However, excessive N (N3) may lead to redundant fruiting branches and unbalanced nutrient allocation: for instance, although the N3 treatment obtained a relatively high number of fruiting branches (14.13) at the boll-opening stage in 2023, not significantly different from N2 (15.11), its value at the flower and boll stage (9.36) was significantly lower than that of N2 (11.04).

Density × nitrogen interactions were significant, especially under the combination of high density and medium nitrogen (D3N2), which produced the highest fruiting branch numbers at the boll-opening stage in both seasons (18.87 in 2022 and 16.33 in 2023). In contrast, nitrogen responses were weak under low density; for example, at the boll-opening stage in 2023, the number of fruiting branches increased only modestly from 11.80 under D1N1 to 13.33 under D1N2. Furthermore, during the flower and boll stage in 2022, the D3N3 treatment produced 11.00 fruiting branches, 25.32% fewer than D3N2 (14.73), suggesting that excessive N under high density may suppress fruiting branch development and impair canopy structural coherence.

### 3.3. Changes in LAI of Cotton Under Different Treatments

Two-year data showed that the leaf area index (LAI) of cotton increased progressively with phenological development, peaked at the flower and boll stage, and then declined slightly thereafter (Table 4).

Regarding density effects, the high-density treatment D3 consistently exhibited a higher LAI at all growth stages. In 2023, D3 reached 4.71 at the flower and boll stage, 13.22% greater than the low-density treatment D1 (4.16); its LAI remained as high as 3.71 at the boll-opening stage, indicating that greater density favors the establishment and maintenance of a robust canopy. Similar trends appeared in 2022, when D3 recorded 4.51 at the flower and boll stage, 9.46% above D1 (4.12).

As for nitrogen level, the medium-N treatment (N2) attained the highest LAI at the flower and boll stage; e.g., in 2022, N2 reached 4.51, significantly exceeding N1 (4.09), and showed the same advantage in 2023. By contrast, the high-N treatment (N3) did not further enhance the LAI and even declined at the boll-opening stage (3.65 in 2023, below the 3.82 of N2), suggesting that excessive N may hasten canopy senescence or reduce resource-use efficiency.

Analysis of the density × nitrogen interaction showed that the D3N2 combination (high density plus medium N) significantly increased the LAI in both years. In 2023, D3N2 reached 4.90 at the flower and boll stage, the highest value recorded, and maintained 3.94 at the boll-opening stage. In contrast, although D3N3 achieved 4.57 at the flower and boll stage in 2022, its LAI fell sharply to 3.57 at the boll-opening stage, indicating that surplus N under high density may not confer a sustained canopy advantage and may accelerate decline. Additionally, nitrogen efficacy was limited under low density; for example, at the boll-opening stage in 2023, the LAI rose from 3.39 under D1N1 to only 3.73 under D1N2, still lower than D3N2 (3.94), demonstrating that low density restricts N responsiveness.

In summary, increasing density coupled with a moderate N supply significantly enhances the stand LAI, optimizes canopy architecture, and thereby augments photosynthetic efficiency and subsequent dry matter accumulation. Excessive N under high density does not necessarily confer additional benefits and may even exert negative effects on canopy structure and physiological activity.

### 3.4. Changes in POD Activity of Cotton Leaves Under Different Treatments

Peroxidase (POD) activity is a key indicator for evaluating the antioxidant capacity and stress resistance of cotton. Data from 2022 to 2023 (Table 5) showed that POD activity followed a “rise–fall” pattern during development, peaking at the flower and boll stage and declining slightly at the boll-opening stage, indicating that the antioxidant system is most active during the vigorous growth phase.

The main effect of planting density showed that high density (D3) significantly increased POD activity. In 2023 at the flower and boll stage, D3 reached 1935.75 U·g^−1^, which was 32.07% higher than the low-density treatment D1 (1465.65 U·g^−1^); a similar pattern was observed in 2022, indicating that high density helps delay functional leaf senescence and strengthen antioxidant defense.

Regarding nitrogen rate, the medium level (N2) performed best at the flower and boll stage in both years, with activity significantly higher than the low-N treatment (N1). Although the high-N treatment (N3) showed relatively high activity during peak growth, its activity declined sharply at the boll-opening stage, implying that excessive nitrogen may accelerate senescence.

The density × nitrogen interaction indicated that the combination of high density and medium nitrogen (D3N2) significantly increased POD activity in both years, outperforming all other combinations.

In summary, POD activity responds strongly to planting density and nitrogen rate. Under short-season direct seeding with once-only irrigation, moderately increasing density combined with a medium nitrogen rate markedly enhances antioxidant enzyme activity, helping delay senescence, improve late-season physiological activity, and stabilize yield.

### 3.5. Changes in MDA Activity of Cotton Leaves Under Different Treatments

Malondialdehyde (MDA) content increased gradually with crop development, peaking at the boll-opening stage, indicating that the degree of membrane lipid peroxidation intensified as plants aged (Table 6).

The main effect of density was significant: at the boll-opening stage in 2023, MDA in the low-density treatment D1 reached 665.54 nmol·g^−1^, 5.16% higher than D2 (632.90 nmol·g^−1^) and 12.43% higher than D3 (591.95 nmol·g^−1^); a similar pattern occurred in 2022, suggesting that higher densities effectively reduce oxidative damage.

Regarding nitrogen, the medium-N treatment (N2) produced the lowest MDA levels in both years; for example, at the boll-opening stage in 2022, MDA was 586.08 nmol·g^−1^ under N2, significantly lower than that under N1 (613.5 nmol·g^−1^), indicating that adequate nitrogen application alleviates membrane damage.

For the density × nitrogen interaction, the D3N2 combination showed the lowest MDA content in both years, outperforming the other treatments. This indicates that high density combined with medium nitrogen alleviates lipid peroxidation and delays senescence. Conversely, MDA remained high under low density; at the boll-opening stage in 2023, D1N1 reached 676.98 nmol·g^−1^, indicating weaker stress tolerance.

Overall, moderately increasing density together with medium nitrogen concentration effectively lowers MDA, stabilizes membrane structure, and delays aging.

### 3.6. Changes in Cotton Yield and Yield Traits Under Different Treatments

Significant differences in boll number per plant and boll weight were detected among treatments, ultimately influencing both seed and lint cotton yields (Table 7). Although lint percentage showed statistical variation between the two seasons, the absolute range was narrow and therefore not a key yield-determining factor.

Across both experimental years, the high-density treatment D3 enhanced crop yield by markedly increasing both boll number per plant and single-boll weight; in 2023, seed cotton yield under D3 reached 2451.63 kg·ha^−1^, 52.81% higher than that of D1 (1604.35 kg·ha^−1^). A comparable pattern occurred in 2022.

With respect to nitrogen rate, the medium level (N2) showed the best performance, producing higher boll number per plant and greater boll weight than both N1 and N3. Consequently, seed and lint cotton yields in 2023 and 2022 under N2 exceeded those under N1 and N3.

For the density × nitrogen interaction, the D3N2 combination achieved the highest yields in both years: seed cotton yield reached 3174.24 kg·ha^−1^ and 2918.90 kg·ha^−1^ and lint cotton yield reached 1297.55 kg·ha^−1^ and 1189.48 kg·ha^−1^ in 2022 and 2023, respectively. These results demonstrate that under the “short-season direct-seeding with once-only irrigation” system, a high-density stand supplied with medium nitrogen maximizes population advantages and yield. In contrast, nitrogen responses were weak at low density; yields of D1N1 and D1N2 lagged behind even D3N1, indicating that although the per-plant boll set improved, the total crop yield remained constrained, highlighting the need for density optimization to achieve high productivity.

### 3.7. Changes in Fiber Quality of Cotton Under Different Treatments

The various treatments exerted minimal influence on fiber quality traits; differences among treatments were minor overall and all metrics remained within the standard range for premium cotton (Table 8).

Regarding stand density, the average length of the upper half under the high-density treatment D3 reached 30.66 mm in 2023, marginally higher than the low-density D1 value of 30.59 mm. Uniformity index, fiber strength, micronaire value, and elongation showed no significant variation, indicating that increased density did not compromise fiber quality.

For the main effect of nitrogen, no significant differences were detected among the N1, N2, and N3 treatments.

Under the density × nitrogen interaction, no significant differences were observed among treatments in either year, suggesting that, in this study, neither stand density nor N rate materially affected fiber quality, which appears to be governed principally by genetic factors.

## 4. Discussion

With increasing constraints on labor and resource inputs, designing cotton production systems that are both resource-efficient and yield-stable has become a major research frontier [18]. Departing from the conventional regime of drip irrigation coupled with multiple fertilizer applications, the short-season direct-seeded cotton strategy, which is characterized by “once irrigation–once fertilization”, streamlines field operations; however, in doing so, it decreases the agronomic window for optimizing plant density and nitrogen supply. Although earlier work highlights that synchronizing plant density with the N rate underpins yield stability and resource-use efficiency in cotton, this interplay remains untested when water is delivered only once [19]. Using two consecutive field trials (2022 and 2023), we unraveled the composite regulatory effects of density and nitrogen within a “short-season direct-seeding + once-only irrigation” framework, furnishing both mechanistic insight and actionable guidance for producers.

A high planting density was the cornerstone of the high-yield canopy architecture assembled in this study. Elevating the density from D1 (30,000 plants·ha^−1^) to D3 (60,000 plants·ha^−1^) markedly increased the plant height, leaf area index (LAI), and number of fruiting branches, revealing that cotton mitigates spatial competition by elongating stems and expanding branch production. This response parallels the observations of Zhang et al. for the Yangtze River Basin [20]. Crucially, we captured the same pattern under a once-only irrigation regime, indicating that density—rather than water availability—primarily governs canopy assembly. After the LAI peaked at the flower and boll stage, plots at D3 retained a superior LAI through the boll-opening stage, underscoring the capacity of a dense canopy to prolong the photosynthetic activity of leaf area. These insights substantiate the development of “water-saving direct-seeded cotton” for arid and semi-arid regions and provide a quantitative foundation for canopy optimization.

Nitrogen modulated canopy performance on top of this density-driven framework. Contrary to reports that escalating N inputs invariably raise cotton yield [21,22], our data show that the high-N rate (N3) accelerated early increases in plant height and the LAI but, by mid-season, sharply elevated malondialdehyde (MDA) and depressed peroxidase (POD) activity. These shifts precipitated an early decline in photosynthetic competence, curtailed fruiting-branch formation, and ultimately eroded yield—a pattern consistent with Boquet’s assertion that excessive N favors vegetative over reproductive growth [23]. Within a single-irrigation context, late-season N top-dressing is impossible; hence, front-loaded N must simultaneously trigger early growth and safeguard subsequent stability. The medium-N regime (N2) achieved this balance: POD activity at the flower and boll stage surpassed all other treatments, MDA remained consistently low, and source strength and organ integrity progressed in concert, culminating in significant gains in productive branches and bolls per plant.

A notable interaction between planting density and nitrogen rate was observed. The D3N2 treatment—high density with moderate nitrogen—consistently yielded better agronomic and physiological outcomes. This may be due to improved early nitrogen uptake and more effective canopy development under high density, while a moderate nitrogen supply potentially limited excessive vegetative growth and maintained a favorable source–sink balance. In contrast, D3N3 (high density, high N) showed signs of oxidative stress later in the season, possibly due to nutrient oversupply and poor canopy ventilation, which might have impaired boll retention. The increased MDA and reduced POD activity support this interpretation. At the other extreme, D1N1 (low density, low N) likely suffered from inadequate resource capture and weak sink development, leading to lower yield. Although parameters such as root competition and nitrogen partitioning were not measured, these findings suggest that planting density and nitrogen rate jointly influence physiological performance through their effects on canopy structure and resource allocation [24].

We show that the yield-forming process is an uninterrupted continuum composed of “early canopy assembly, mid-season stability, and late-season assimilate conversion”. The LAI and fruiting-branch architecture are primarily built between the bud stage and the flower and boll stage; POD and MDA trajectories index the stand’s stress buffer capacity and the lifespan of functional organs; and the ultimate conversion efficiency, captured by bolls per plant and boll weight, determines the final yield outcome. Whereas previous work has tended to correlate end-point yield metrics with a single factor [25,26], our whole-season coupling of canopy architecture, physiological sustainment, and yield composition demonstrates that yield is not the additive output of isolated determinants but the emergent property of stage-wise dynamic regulation.

Operating under a one-time “water-plus-nitrogen” delivery regime introduces a rigid resource allocation framework: the entire quota of water and N is front-loaded at emergence and must carry the crop through to boll opening, thereby requiring precise early canopy construction and leaving little room for mid- or late-season fine-tuning. The superiority of D3N2—manifested as sustained LAI, low lipid peroxidation (MDA), abundant fruiting branches, and high yield—is therefore rooted in its optimal resource deployment pattern at canopy initiation. This trajectory parallels the “robust early root establishment followed by maintenance of functional structure through mid and late season” strategy described by Hunsaker and Bronson for drip-irrigated cotton in arid zones [27].

Importantly, no appreciable deterioration in fiber quality was observed under the strongly modulated canopy architecture. In D3N2 plots, metrics such as fiber length, fiber strength, and uniformity index remained stable, suggesting that a high-density, moderate-N regime safeguards—rather than compromises—fiber development by sustaining a consistent source supply. This agrees with Watts et al., who reported that the canopy’s ecological balance exerts a stronger influence on fiber quality than nitrogen dosage alone [28]. Thus, density–nitrogen synergy elevates yield without sacrificing lint marketability, highlighting its promise for broad adoption.

Collectively, our results establish—within a “one-irrigation, one-fertilization” framework—a tripartite regulatory paradigm for short-season cotton: canopy assembly is governed primarily by planting density, physiological equilibrium is fine-tuned by nitrogen, and their interaction ultimately shapes yield. A robust canopy produced under high density lays the groundwork, and moderate N input preserves metabolic stability and allocative efficiency; the concerted action of the two secures both early-season drive and late-season steadiness in yield expression. In water-limited systems with streamlined management, this scheme furnishes a sound agronomic blueprint for producing short-season direct-seeded cotton that is efficient, productive, and of superior quality.

## 5. Conclusions

Based on two years (2022–2023) of field trials under a once-only irrigation regime, this study evaluated the interactive effects of three planting densities (30,000, 45,000, and 60,000 plants·ha^−1^) and three nitrogen rates (150, 180, and 210 kg·ha^−1^) in short-season direct-seeded cotton.

The results showed that a higher density (60,000 plants·ha^−1^) significantly improved canopy architecture by increasing the plant height, leaf area, and fruiting-site capacity per unit area. Meanwhile, a moderate nitrogen rate (180 kg·ha^−1^) appeared to sustain antioxidant activity (e.g., higher POD) and limit oxidative damage (e.g., lower MDA), potentially delaying senescence and improving source–sink coordination during boll development.

The combination of high density and moderate nitrogen (D3N2) consistently produced the highest seed cotton and lint yields in both years. This likely resulted from optimized canopy development, improved stress resilience, and efficient resource allocation early in the season. In contrast, excessive nitrogen (210 kg·ha^−1^) promoted early vigor but may have accelerated senescence and reduced boll retention under limited water conditions.

Notably, fiber quality traits such as length, strength, and uniformity were not negatively affected by yield-enhancing treatments, supporting the viability of a high-yield, high-quality system.

Overall, the study suggests that under simplified management with one-time irrigation and fertilization, adopting 60,000 plants·ha^−1^ with 180 kg·N·ha^−1^ is a promising strategy to balance yield, fiber quality, and input efficiency. These findings provide actionable guidance for intensifying short-season cotton systems in labor- and water-limited environments.

## Figures and Tables

**Table 1:** The physicochemical properties of soil in 0–20 cm tillage layer of experimental sites in 2022 and 2023.

Year	Organic Material g/kg	pH Value	Total Nitrogen (g/kg)	Alkaline Nitrogen Decomposition (mg/kg)	Effective Phosphorus (mg/kg)	Quick-Acting Potassium (mg/kg)
2022	24.94	5.73	1.49	152.15	104.76	150
2023	25.70	5.86	1.55	153.34	126.13	120

**Table 2:** Effect of different treatments on cotton plant height (cm).

2022 Year	Treatment	Seedling Stage	Bud Stage	Flower and Boll Stage	Boll-Opening Stage
Density	D1	24.58 ± 1.60 b	46.04 ± 1.06 b	96.80 ± 1.97 b	111.82 ± 4.69 b
	D2	24.80 ± 1.25 b	52.49 ± 2.15 ab	100.44 ± 1.51 ab	121.24 ± 5.73 a
	D3	29.78 ± 2.54 a	59.87 ± 3.54 a	110.29 ± 4.20 a	123.60 ± 3.92 a
Nitrogen	N1	24.91 ± 2.73 b	50.93 ± 4.67 b	100.31 ± 5.74 a	114.78 ± 6.80 b
	N2	28.53 ± 3.41 a	54.84 ± 6.79 a	104.91 ± 7.99 a	123.16 ± 5.86 a
	N3	25.71 ± 1.67 b	52.62 ± 7.03 ab	102.31 ± 4.97 a	118.73 ± 6.12 a
Density × Nitrogen	D1N1	22.60 ± 0.20 f	45.73 ± 0.99 d	94.67 ± 1.80 d	107.00 ± 1.71 d
	D1N2	26.20 ± 0.35 d	47.20 ± 0.53 d	98.13 ± 1.15 cd	116.73 ± 2.69 bc
	D1N3	24.93 ± 0.23 de	45.20 ± 0.20 d	97.60 ± 0.72 cd	111.73 ± 2.61 cd
	D2N1	23.67 ± 0.23 ef	51.00 ± 1.91 c	99.07 ± 0.50 c	115.27 ± 1.33 c
	D2N2	26.40 ± 0.35 cd	54.93 ± 0.92 b	101.47 ± 1.81 c	125.87 ± 5.08 a
	D2N3	24.33 ± 0.12 e	51.53 ± 0.61 c	100.80 ± 1.00 c	122.60 ± 3.90 a
	D3N1	28.47 ± 0.58 b	56.07 ± 1.63 b	107.20 ± 2.60 b	122.07 ± 3.04 a
	D3N2	33.00 ± 1.22 a	62.40 ± 3.12 a	115.13 ± 2.64 a	126.87 ± 3.30 a
	D3N3	27.87 ± 0.64 bc	61.13 ± 2.02 a	108.53 ± 1.62 b	121.87 ± 4.16 ab
2023 Year					
Density	D1	24.29 ± 2.30 b	45.80 ± 1.02 c	93.96 ± 1.46 c	108.00 ± 3.82 c
	D2	24.71 ± 1.52 b	53.13 ± 1.64 b	96.33 ± 1.79 b	117.31 ± 3.27 b
	D3	29.80 ± 2.64 a	57.60 ± 2.82 a	99.96 ± 4.51 a	121.82 ± 4.26 a
Nitrogen	N1	24.40 ± 3.04 c	50.44 ± 4.35 c	94.18 ± 1.70 c	112.53 ± 6.22 b
	N2	28.87 ± 3.34 a	54.33 ± 6.04 a	99.69 ± 4.57 a	120.16 ± 6.62 a
	N3	25.53 ± 1.80 b	51.76 ± 5.23 b	96.38 ± 2.27 b	114.44 ± 6.10 b
Density × Nitrogen	D1N1	21.87 ± 0.90 g	45.13 ± 0.42 e	92.20 ± 0.35 e	104.87 ± 2.53 e
	D1N2	27.00 ± 0.53 bc	47.07 ± 0.23 e	95.20 ± 0.87 cde	112.47 ± 1.53 d
	D1N3	24.00 ± 0.35 ef	45.20 ± 0.60 e	94.47 ± 0.58 de	106.67 ± 1.55 e
	D2N1	23.00 ± 0.35 fg	51.27 ± 0.50 d	94.47 ± 0.58 de	114.33 ± 1.72 cd
	D2N2	26.33 ± 0.50 cd	55.00 ± 0.20 bc	98.47 ± 0.46 bc	120.73 ± 2.66 b
	D2N3	24.80 ± 0.72 de	53.13 ± 0.23 cd	96.07 ± 0.23 bcd	116.87 ± 1.21 bcd
	D3N1	28.33 ± 0.50 b	54.93 ± 1.33 bc	95.87 ± 0.90 bcd	118.40 ± 0.87 bc
	D3N2	33.27 ± 0.42 a	60.93 ± 0.81 a	105.40 ± 1.04 a	127.27 ± 1.03 a
	D3N3	27.80 ± 0.53 bc	56.93 ± 1.14 b	98.60 ± 2.69 b	119.80 ± 1.59 b

Different lowercase letters indicate significant differences between treatments under the same fertility period according to Duncan’s test (*p* < 0.05).

**Table 3:** Effect of different treatments on the number of fruiting branches in cotton.

2022 Year	Treatment	Bud Stage	Flower and Boll Stage	Boll-Opening Stage
Density	D1	4.47 ± 0.30 b	9.04 ± 1.35 b	14.62 ± 2.02 a
	D2	6.24 ± 0.60 a	8.84 ± 0.78 b	15.47 ± 1.89 a
	D3	6.76 ± 1.00 a	12.60 ± 1.72 a	16.64 ± 1.80 a
Nitrogen	N1	5.16 ± 0.79 a	9.36 ± 2.10 b	14.02 ± 1.25 b
	N2	6.18 ± 1.15 a	11.69 ± 2.36 a	17.89 ± 1.01 a
	N3	6.13 ± 1.42 a	9.44 ± 1.24 a	14.82 ± 1.14 b
Density × Nitrogen	D1N1	4.20 ± 0.20 d	7.60 ± 0.35 e	13.20 ± 0.87 d
	D1N2	4.67 ± 0.23 d	10.53 ± 0.76 c	17.13 ± 0.95 ab
	D1N3	4.53 ± 0.31 d	9.00 ± 0.35 de	13.53 ± 0.64 cd
	D2N1	5.67 ± 0.31 c	8.40 ± 0.35 de	13.47 ± 0.58 cd
	D2N2	6.93 ± 0.12 ab	9.80 ± 0.40 cd	17.67 ± 0.61 a
	D2N3	6.13 ± 0.31 bc	8.33 ± 0.31 e	15.27 ± 0.58 bc
	D3N1	5.60 ± 0.53 c	12.07 ± 0.61 b	15.40 ± 0.92 bc
	D3N2	6.93 ± 0.23 ab	14.73 ± 0.50 a	18.87 ± 0.64 a
	D3N3	7.73 ± 0.42 a	11.00 ± 0.40 bc	15.67 ± 0.76 b
2023 Year				
Density	D1	4.13 ± 0.37 c	8.62 ± 1.30 b	12.58 ± 0.71 b
	D2	5.76 ± 0.56 b	8.93 ± 1.14 b	14.49 ± 1.84 a
	D3	6.53 ± 0.76 a	11.53 ± 0.83 a	15.00 ± 1.04 a
Nitrogen	N1	5.00 ± 0.81 a	8.67 ± 1.76 b	12.82 ± 1.26 b
	N2	6.11 ± 1.33 a	11.04 ± 1.21 a	15.11 ± 1.44 a
	N3	5.29 ± 1.12 a	9.36 ± 1.25 b	14.13 ± 1.42 a
Density × Nitrogen	D1N1	4.07 ± 0.49 d	7.27 ± 0.13 f	11.80 ± 0.18 e
	D1N2	4.47 ± 0.13 d	10.20 ± 0.34 c	13.33 ± 0.36 cd
	D1N3	3.87 ± 0.20 d	8.40 ± 0.16 de	12.60 ± 0.16 de
	D2N1	5.20 ± 0.20 c	7.80 ± 0.24 ef	12.20 ± 0.22 de
	D2N2	6.40 ± 0.29 b	10.33 ± 0.51 bc	15.66 ± 0.63 ab
	D2N3	5.67 ± 0.14 c	8.67 ± 0.13 d	15.60 ± 1.07 ab
	D3N1	5.80 ± 0.24 bc	11.00 ± 0.26 b	14.47 ± 0.20 bc
	D3N2	7.47 ± 0.13 a	12.60 ± 0.28 a	16.33 ± 0.36 a
	D3N3	6.30 ± 0.31 b	11.00 ± 0.28 b	14.20 ± 0.21 c

Different lowercase letters indicate significant differences between treatments under the same fertility period according to Duncan’s test (*p* < 0.05).

**Table 4:** Effect of different treatments on leaf area index of cotton.

2022 Year	Treatment	Seedling Stage	Bud Stage	Flower and Boll Stage	Boll-Opening Stage
Density	D1	0.92 ± 0.10 b	3.04 ± 0.12 c	4.12 ± 0.18 c	3.52 ± 0.15 a
	D2	1.17 ± 0.14 a	3.17 ± 0.12 b	4.38 ± 0.21 b	3.58 ± 0.15 a
	D3	1.20 ± 0.17 a	3.32 ± 0.19 a	4.51 ± 0.22 a	3.61 ± 0.15 a
Nitrogen	N1	1.02 ± 0.14 b	3.10 ± 0.15 b	4.09 ± 0.16 c	3.41 ± 0.09 c
	N2	1.25 ± 0.20 a	3.32 ± 0.20 a	4.51 ± 0.20 a	3.73 ± 0.07 a
	N3	1.03 ± 0.12 b	3.11 ± 0.11 b	4.41 ± 0.20 b	3.57 ± 0.08 b
Density × Nitrogen	D1N1	0.86 ± 0.10 d	2.94 ± 0.09 c	3.90 ± 0.02 e	3.36 ± 0.11 e
	D1N2	1.00 ± 0.08 cd	3.15 ± 0.12 bc	4.29 ± 0.06 c	3.68 ± 0.05 abc
	D1N3	0.89 ± 0.09 d	3.04 ± 0.08 bc	4.17 ± 0.05 cd	3.52 ± 0.05 bcde
	D2N1	1.05 ± 0.08 cd	3.12 ± 0.08 bc	4.13 ± 0.04 d	3.40 ± 0.06 de
	D2N2	1.33 ± 0.10 ab	3.27 ± 0.09 ab	4.53 ± 0.04 b	3.72 ± 0.07 ab
	D2N3	1.13 ± 0.06 bc	3.12 ± 0.14 bc	4.49 ± 0.12 b	3.62 ± 0.09 abcd
	D3N1	1.14 ± 0.08 bc	3.24 ± 0.08 b	4.24 ± 0.05 cd	3.47 ± 0.07 cde
	D3N2	1.41 ± 0.08 a	3.54 ± 0.10 a	4.72 ± 0.07 a	3.78 ± 0.07 a
	D3N3	1.06 ± 0.07 cd	3.16 ± 0.07 bc	4.57 ± 0.05 b	3.57 ± 0.08 abcde
2023 Year					
Density	D1	1.42 ± 0.10 c	3.20 ± 0.12 c	4.16 ± 0.14 c	3.57 ± 0.16 b
	D2	1.49 ± 0.11 b	3.50 ± 0.11 b	4.56 ± 0.16 b	3.65 ± 0.15 ab
	D3	1.62 ± 0.11 a	3.69 ± 0.17 a	4.71 ± 0.17 a	3.71 ± 0.20 a
Nitrogen	N1	1.41 ± 0.11 c	3.33 ± 0.22 c	4.32 ± 0.22 c	3.46 ± 0.08 c
	N2	1.63 ± 0.11 a	3.61 ± 0.27 a	4.65 ± 0.28 a	3.82 ± 0.12 a
	N3	1.50 ± 0.07 b	3.45 ± 0.18 b	4.47 ± 0.27 b	3.65 ± 0.06 b
Density × Nitrogen	D1N1	1.30 ± 0.08 e	3.06 ± 0.07 e	4.04 ± 0.06 f	3.39 ± 0.05 e
	D1N2	1.52 ± 0.03 bc	3.29 ± 0.05 d	4.30 ± 0.07 de	3.73 ± 0.09 abc
	D1N3	1.44 ± 0.03 cd	3.24 ± 0.04 d	4.15 ± 0.13 ef	3.59 ± 0.06 bcde
	D2N1	1.39 ± 0.06 de	3.39 ± 0.04 cd	4.41 ± 0.07 cd	3.47 ± 0.05 de
	D2N2	1.62 ± 0.01 b	3.64 ± 0.02 b	4.74 ± 0.08 b	3.79 ± 0.08 ab
	D2N3	1.47 ± 0.04 cd	3.47 ± 0.03 c	4.55 ± 0.11 c	3.68 ± 0.06 bcd
	D3N1	1.52 ± 0.07 bc	3.53 ± 0.07 bc	4.52 ± 0.03 c	3.52 ± 0.08 cde
	D3N2	1.75 ± 0.04 a	3.89 ± 0.12 a	4.90 ± 0.08 a	3.94 ± 0.10 a
	D3N3	1.58 ± 0.03 b	3.65 ± 0.05 b	4.72 ± 0.05 b	3.67 ± 0.04 bcd

Different lowercase letters indicate significant differences between treatments under the same fertility period according to Duncan’s test (*p* < 0.05).

**Table 5:** Effect of different treatments on POD activity (U·g^−1^) of cotton leaves.

2022 Year	Treatment	Seedling Stage	Bud Stage	Flower and Boll Stage	Boll-Opening Stage
Density	D1	458.14 ± 18.51 c	1076.08 ± 125.39 c	1349.32 ± 50.57 c	889.49 ± 20.57 c
	D2	495.43 ± 24.74 b	1237.84 ± 127.16 b	1664.99 ± 188.83 b	951.63 ± 23.23 b
	D3	517.01 ± 27.87 a	1431.31 ± 143.20 a	1818.93 ± 213.95 a	1024.83 ± 76.77 a
Nitrogen	N1	464.78 ± 23.86 b	1141.92 ± 162.08 b	1478.41 ± 138.05 c	931.63 ± 52.82 b
	N2	508.99 ± 29.34 a	1401.18 ± 174.10 a	1785.93 ± 302.56 a	993.79 ± 99.91 a
	N3	496.81 ± 33.54 a	1202.14 ± 159.88 b	1568.90 ± 214.82 b	940.53 ± 43.22 b
Density × Nitrogen	D1N1	441.60 ± 12.24 d	968.93 ± 23.67 f	1313.36 ± 51.66 d	871.36 ± 19.76 d
	D1N2	476.71 ± 11.14 cd	1223.53 ± 69.71 cd	1398.67 ± 2.65 d	906.52 ± 14.25 bcd
	D1N3	456.11 ± 12.77 d	1035.79 ± 72.14 ef	1335.92 ± 41.13 d	890.60 ± 13.03 cd
	D2N1	467.75 ± 20.51 cd	1148.44 ± 126.27 de	1540.64 ± 78.28 c	936.78 ± 23.36 bcd
	D2N2	511.45 ± 11.54 abc	1377.12 ± 22.64 b	1905.13 ± 69.81 b	971.23 ± 15.60 bc
	D2N3	507.08 ± 12.51 abc	1187.98 ± 58.36 cd	1549.20 ± 42.71 c	946.88 ± 20.68 bcd
	D3N1	485.00 ± 16.55 bcd	1308.38 ± 45.39 bc	1581.23 ± 70.05 c	986.75 ± 13.29 b
	D3N2	538.80 ± 16.74 a	1602.89 ± 81.47 a	2054.00 ± 85.05 a	1103.63 ± 95.91 a
	D3N3	527.23 ± 12.18 ab	1382.64 ± 54.33 b	1821.57 ± 57.56 b	984.12 ± 14.95 bc
2023 Year					
Density	D1	470.65 ± 19.32 c	1074.83 ± 125.49 c	1465.65 ± 95.91 c	939.05 ± 57.52 b
	D2	494.74 ± 20.55 b	1168.77 ± 128.26 b	1691.58 ± 191.91 b	976.32 ± 52.76 b
	D3	523.65 ± 32.81 a	1369.03 ± 95.62 a	1935.75 ± 254.24 a	1056.40 ± 53.83 a
Nitrogen	N1	472.80 ± 19.02 c	1096.75 ± 145.35 c	1512.95 ± 143.63 c	941.16 ± 59.32 b
	N2	521.29 ± 30.74 a	1341.16 ± 111.78 a	1911.56 ± 291.66 a	1034.15 ± 80.53 a
	N3	494.95 ± 28.72 b	1174.73 ± 153.18 b	1668.47 ± 196.29 b	996.46 ± 46.40 a
Density × Nitrogen	D1N1	453.34 ± 16.55 d	976.14 ± 16.91 d	1367.67 ± 21.10 e	875.55 ± 11.54 c
	D1N2	490.61 ± 7.84 bcd	1234.67 ± 50.80 bc	1575.86 ± 19.74 cd	966.48 ± 61.87 bc
	D1N3	467.98 ± 10.03 d	1013.68 ± 39.79 d	1453.43 ± 55.84 de	975.13 ± 12.03 bc
	D2N1	478.15 ± 9.55 cd	1031.57 ± 62.06 d	1489.73 ± 63.96 de	939.53 ± 15.32 bc
	D2N2	517.81 ± 16.04 abc	1311.30 ± 26.30 b	1919.15 ± 57.13 b	1022.60 ± 71.24 ab
	D2N3	488.25 ± 8.12 cd	1163.45 ± 50.13 c	1665.87 ± 13.12 c	966.83 ± 21.09 bc
	D3N1	486.91 ± 13.15 cd	1282.54 ± 19.84 bc	1681.46 ± 54.05 c	1008.39 ± 21.64 ab
	D3N2	555.43 ± 16.81 a	1477.50 ± 21.80 a	2239.66 ± 77.52 a	1113.37 ± 23.47 a
	D3N3	528.62 ± 16.53 ab	1347.06 ± 78.19 b	1886.12 ± 101.94 b	1047.43 ± 46.10 ab

Different lowercase letters indicate significant differences between treatments under the same fertility period according to Duncan’s test (*p* < 0.05).

**Table 6:** Effect of different treatments on the MDA content (nmol·g^−1^) of cotton leaves.

2022 Year	Treatment	Seedling Stage	Bud Stage	Flower and Boll Stage	Boll-Opening Stage
Density	D1	287.24 ± 32.37 a	448.92 ± 15.66 a	506.75 ± 10.15 a	619.33 ± 13.65 a
	D2	251.37 ± 7.89 b	431.74 ± 18.51 b	490.17 ± 13.40 b	613.42 ± 11.22 a
	D3	231.36 ± 8.46 c	410.88 ± 16.42 c	479.88 ± 12.49 c	568.49 ± 16.77 b
Nitrogen	N1	268.20 ± 38.04 a	449.91 ± 16.92 a	503.65 ± 12.47 a	613.50 ± 23.50 a
	N2	244.50 ± 19.51 b	415.59 ± 17.09 c	481.62 ± 14.41 c	586.08 ± 28.11 c
	N3	257.27 ± 28.99 ab	426.04 ± 19.98 b	491.52 ± 14.66 b	601.66 ± 23.76 b
Density × Nitrogen	D1N1	307.83 ± 44.29 a	464.64 ± 10.60 a	517.27 ± 4.78 a	630.96 ± 9.91 a
	D1N2	265.46 ± 10.53 abc	433.69 ± 8.41 bc	496.23 ± 5.98 bc	603.73 ± 5.88 bc
	D1N3	288.41 ± 27.71 ab	448.44 ± 8.87 ab	506.73 ± 4.59 ab	623.30 ± 4.64 ab
	D2N1	256.58 ± 8.17 bc	453.62 ± 12.92 ab	500.44 ± 9.86 ab	625.06 ± 12.63 ab
	D2N2	244.75 ± 5.93 bc	416.47 ± 5.29 cde	479.43 ± 12.08 cde	605.30 ± 4.74 bc
	D2N3	252.79 ± 6.15 bc	425.13 ± 6.56 cd	490.66 ± 11.97 bcd	609.92 ± 1.17 ab
	D3N1	240.18 ± 4.72 c	431.48 ± 3.16 bc	493.25 ± 6.76 bcd	584.50 ± 5.63 cd
	D3N2	223.30 ± 6.50 c	396.60 ± 6.05 e	469.19 ± 9.47 e	549.21 ± 6.68 e
	D3N3	230.62 ± 2.57 c	404.56 ± 5.40 de	477.18 ± 6.16 de	571.76 ± 9.50 de
2023 Year					
Density	D1	329.67 ± 20.72 a	460.94 ± 13.27 a	524.92 ± 13.35 a	665.54 ± 11.93 a
	D2	285.00 ± 21.88 b	437.30 ± 16.56 b	503.27 ± 15.85 b	632.90 ± 14.77 b
	D3	239.92 ± 14.19 c	426.81 ± 18.48 c	475.67 ± 15.19 c	591.95 ± 21.61 c
Nitrogen	N1	302.35 ± 44.89 a	457.37 ± 16.77 a	515.79 ± 19.02 a	645.59 ± 27.84 a
	N2	263.57 ± 36.70 c	425.64 ± 18.56 c	486.08 ± 21.52 c	613.02 ± 38.47 c
	N3	288.66 ± 37.52 b	442.04 ± 16.94 b	501.99 ± 26.65 b	631.78 ± 32.11 b
Density × Nitrogen	D1N1	348.66 ± 11.16 a	473.49 ± 5.77 a	536.56 ± 6.19 a	676.98 ± 7.50 a
	D1N2	305.75 ± 9.66 b	446.71 ± 10.53 bcd	509.78 ± 9.02 bc	655.35 ± 6.87 abc
	D1N3	334.59 ± 7.96 a	462.63 ± 3.95 ab	528.43 ± 5.33 ab	664.31 ± 10.56 ab
	D2N1	310.68 ± 9.00 b	455.23 ± 4.74 abc	516.80 ± 5.00 ab	644.57 ± 12.39 bc
	D2N2	262.80 ± 5.20 cd	420.61 ± 9.24 ef	485.63 ± 6.16 de	616.27 ± 4.26 de
	D2N3	281.53 ± 7.81 c	436.07 ± 9.33 cde	507.39 ± 13.23 bc	637.85 ± 6.67 cd
	D3N1	249.87 ± 3.18 d	443.40 ± 19.49 bcde	494.01 ± 4.81 cd	615.21 ± 5.14 de
	D3N2	222.18 ± 5.43 e	409.62 ± 9.66 f	462.84 ± 8.89 f	567.45 ± 5.94 f
	D3N3	247.71 ± 7.34 d	427.42 ± 6.01 def	470.16 ± 4.91 ef	593.18 ± 9.58 e

Different lowercase letters indicate significant differences between treatments under the same fertility period according to Duncan’s test (*p* < 0.05).

**Table 7:** Effects of different treatments on yield traits and yield of cotton.

2022 Year	Treatment	Boll Number per Plant/Pieces	Boll Weight /g	Lint Percentage /%	Seed CottonYield /(kg·ha^−1^)	Lint Yield /(kg·ha^−1^)
Density	D1	19.56 ± 1.42 c	3.34 ± 0.25 c	40.68 ± 0.04 c	1674.44 ± 238.29 c	681.18 ± 97.25 c
	D2	23.09 ± 0.95 b	3.53 ± 0.15 b	40.73 ± 0.05 b	2082.73 ± 164.00 b	848.28 ± 66.91 b
	D3	27.38 ± 2.52 a	3.90 ± 0.26 a	40.79 ± 0.07 a	2736.83 ± 432.45 a	1116.55 ± 177.95 a
Nitrogen	N1	21.64 ± 2.82 c	3.38 ± 0.26 b	40.70 ± 0.05 b	1879.18 ± 372.43 c	764.98 ± 152.31 c
	N2	25.18 ± 3.95 a	3.79 ± 0.32 a	40.78 ± 0.08 a	2457.86 ± 587.75 a	1002.57 ± 241.69 a
	N3	23.20 ± 3.63 b	3.61 ± 0.27 a	40.72 ± 0.05 b	2156.96 ± 494.25 b	878.46 ± 201.87 b
Density × Nitrogen	D1N1	18.20 ± 0.20 e	3.10 ± 0.20 d	40.65 ± 0.03 b	1439.05 ± 101.11 d	584.92 ± 40.93 e
	D1N2	21.27 ± 0.76 d	3.57 ± 0.21 bc	40.71 ± 0.03 b	1936.30 ± 172.03 bc	788.24 ± 69.61 cd
	D1N3	19.20 ± 0.40 e	3.37 ± 0.06 cd	40.68 ± 0.06 b	1647.98 ± 17.38 cd	670.39 ± 6.32 de
	D2N1	22.20 ± 0.53 d	3.40 ± 0.10 cd	40.71 ± 0.05 b	1924.91 ± 80.03 bc	783.64 ± 32.82 cd
	D2N2	24.20 ± 0.40 c	3.67 ± 0.15 bc	40.74 ± 0.05 b	2263.04 ± 112.97 b	921.93 ± 44.91 c
	D2N3	22.87 ± 0.31 cd	3.53 ± 0.06 bcd	40.74 ± 0.05 b	2060.23 ± 39.04 bc	839.28 ± 16.95 c
	D3N1	24.53 ± 0.83 c	3.63 ± 0.06 bc	40.75 ± 0.03 b	2273.58 ± 105.60 b	926.39 ± 42.46 c
	D3N2	30.07 ± 1.22 a	4.13 ± 0.25 a	40.88 ± 0.02 a	3174.24 ± 323.53 a	1297.55 ± 132.71 a
	D3N3	27.53 ± 0.42 b	3.93 ± 0.15 ab	40.75 ± 0.02 b	2762.67 ± 149.48 a	1125.70 ± 61.14 b
2023 Year						
Density	D1	18.36 ± 1.02 c	3.42 ± 0.14 c	40.61 ± 0.03 c	1604.35 ± 147.01 c	651.60 ± 60.23 c
	D2	19.99 ± 1.27 b	3.76 ± 0.15 b	40.68 ± 0.04 b	1917.43 ± 186.29 b	780.05 ± 76.55 b
	D3	23.16 ± 2.59 a	4.12 ± 0.33 a	40.72 ± 0.04 a	2451.63 ± 453.85 a	998.51 ± 185.58 a
Nitrogen	N1	18.73 ± 1.35 c	3.57 ± 0.22 c	40.63 ± 0.05 c	1709.52 ± 219.81 c	694.70 ± 90.03 c
	N2	22.33 ± 2.89 a	3.96 ± 0.38 a	40.71 ± 0.05 a	2277.04 ± 517.06 a	927.11 ± 211.52 a
	N3	20.43 ± 2.30 b	3.78 ± 0.38 b	40.68 ± 0.06 b	1986.85 ± 422.39 b	808.36 ± 172.94 b
Density × Nitrogen	D1N1	17.33 ± 0.46 g	3.30 ± 0.10 e	40.58 ± 0.02 e	1457.92 ± 22.47 f	591.58 ± 9.25 f
	D1N2	19.53 ± 0.31 def	3.57 ± 0.06 cde	40.65 ± 0.02 cd	1776.84 ± 55.53 de	722.23 ± 22.84 de
	D1N3	18.20 ± 0.40 fg	3.40 ± 0.10 de	40.61 ± 0.02 de	1578.28 ± 71.06 ef	641.00 ± 29.07 ef
	D2N1	18.67 ± 0.31 efg	3.63 ± 0.12 bcd	40.64 ± 0.01 cd	1729.92 ± 78.28 de	703.04 ± 31.93 de
	D2N2	21.47 ± 0.31 c	3.90 ± 0.10 b	40.72 ± 0.03 ab	2135.37 ± 84.89 c	869.61 ± 35.20 c
	D2N3	19.83 ± 0.59 de	3.73 ± 0.12 bc	40.67 ± 0.02 c	1887.00 ± 10.65 d	767.51 ± 4.61 d
	D3N1	20.20 ± 0.87 cd	3.77 ± 0.06 bc	40.68 ± 0.01 bc	1940.72 ± 104.28 cd	789.48 ± 42.23 cd
	D3N2	26.00 ± 0.53 a	4.40 ± 0.20 a	40.75 ± 0.03 a	2918.90 ± 189.05 a	1189.48 ± 77.64 a
	D3N3	23.27 ± 0.76 b	4.20 ± 0.26 a	40.74 ± 0.02 a	2495.26 ± 235.38 b	1016.57 ± 95.99 b

Different lowercase letters indicate significant differences between treatments under the same fertility period according to Duncan’s test (*p* < 0.05).

**Table 8:** Effect of different treatments on fiber quality of cotton.

2022 Year	Treatment	Average Length of Upper Half /mm	Uniformity Index /%	Fiber Strength /cN·tex^−1^	Micronaire Value	Elongation Rate/%
Density	D1	30.57 ± 0.22 b	86.69 ± 0.14 a	32.55 ± 0.23 a	5.26 ± 0.07 a	6.74 ± 0.08 a
	D2	30.92 ± 0.12 a	86.73 ± 0.12 a	32.55 ± 0.17 a	5.24 ± 0.07 a	6.76 ± 0.05 a
	D3	30.86 ± 0.18 a	86.76 ± 0.08 a	32.55 ± 0.15 a	5.30 ± 0.06 a	6.76 ± 0.06 a
Nitrogen	N1	30.68 ± 0.26 a	86.77 ± 0.08 a	32.55 ± 0.21 a	5.26 ± 0.07 a	6.74 ± 0.07 a
	N2	30.87 ± 0.22 a	86.71 ± 0.15 a	32.52 ± 0.20 a	5.29 ± 0.05 a	6.74 ± 0.07 a
	N3	30.80 ± 0.19 a	86.70 ± 0.10 a	32.58 ± 0.13 a	5.25 ± 0.08 a	6.78 ± 0.04 a
Density × Nitrogen	D1N1	30.46 ± 0.28 b	86.79 ± 0.06 a	32.50 ± 0.35 a	5.27 ± 0.05 a	6.69 ± 0.08 a
	D1N2	30.65 ± 0.21 ab	86.63 ± 0.20 a	32.56 ± 0.29 a	5.27 ± 0.02 a	6.75 ± 0.10 a
	D1N3	30.61 ± 0.20 ab	86.65 ± 0.10 a	32.60 ± 0.06 a	5.23 ± 0.12 a	6.80 ± 0.03 a
	D2N1	30.90 ± 0.13 ab	86.83 ± 0.09 a	32.53 ± 0.18 a	5.25 ± 0.09 a	6.78 ± 0.04 a
	D2N2	30.95 ± 0.17 ab	86.71 ± 0.13 a	32.46 ± 0.20 a	5.28 ± 0.06 a	6.71 ± 0.02 a
	D2N3	30.92 ± 0.09 ab	86.64 ± 0.05 a	32.65 ± 0.11 a	5.21 ± 0.05 a	6.78 ± 0.07 a
	D3N1	30.69 ± 0.16 ab	86.70 ± 0.02 a	32.64 ± 0.05 a	5.26 ± 0.09 a	6.76 ± 0.07 a
	D3N2	31.01 ± 0.13 a	86.78 ± 0.11 a	32.53 ± 0.18 a	5.33 ± 0.04 a	6.75 ± 0.09 a
	D3N3	30.89 ± 0.06 ab	86.79 ± 0.05 a	32.48 ± 0.17 a	5.30 ± 0.03 a	6.77 ± 0.03 a
2023 Year						
Density	D1	30.59 ± 0.22 a	86.61 ± 0.19 a	32.52 ± 0.19 a	5.29 ± 0.06 a	6.74 ± 0.07 a
	D2	30.79 ± 0.19 a	86.63 ± 0.19 a	32.54 ± 0.24 a	5.27 ± 0.07 a	6.73 ± 0.05 a
	D3	30.66 ± 0.11 a	86.60 ± 0.12 a	32.70 ± 0.13 a	5.34 ± 0.07 a	6.73 ± 0.04 a
Nitrogen	N1	30.65 ± 0.25 a	86.57 ± 0.17 a	32.61 ± 0.20 a	5.30 ± 0.09 a	6.73 ± 0.06 a
	N2	30.65 ± 0.14 a	86.62 ± 0.22 a	32.54 ± 0.22 a	5.29 ± 0.07 a	6.74 ± 0.06 a
	N3	30.75 ± 0.18 a	86.66 ± 0.10 a	32.61 ± 0.20 a	5.30 ± 0.05 a	6.72 ± 0.05 a
Density × Nitrogen	D1N1	30.47 ± 0.27 a	86.60 ± 0.16 a	32.59 ± 0.07 a	5.29 ± 0.06 a	6.73 ± 0.09 a
	D1N2	30.64 ± 0.20 a	86.60 ± 0.34 a	32.45 ± 0.12 a	5.26 ± 0.05 a	6.75 ± 0.09 a
	D1N3	30.67 ± 0.20 a	86.64 ± 0.09 a	32.51 ± 0.32 a	5.30 ± 0.07 a	6.74 ± 0.08 a
	D2N1	30.87 ± 0.21 a	86.57 ± 0.25 a	32.50 ± 0.29 a	5.24 ± 0.09 a	6.76 ± 0.06 a
	D2N2	30.70 ± 0.14 a	86.68 ± 0.21 a	32.51 ± 0.31 a	5.25 ± 0.07 a	6.71 ± 0.03 a
	D2N3	30.82 ± 0.24 a	86.66 ± 0.15 a	32.61 ± 0.18 a	5.32 ± 0.03 a	6.71 ± 0.05 a
	D3N1	30.62 ± 0.06 a	86.53 ± 0.13 a	32.73 ± 0.16 a	5.38 ± 0.06 a	6.72 ± 0.04 a
	D3N2	30.61 ± 0.11 a	86.59 ± 0.14 a	32.67 ± 0.20 a	5.35 ± 0.06 a	6.75 ± 0.07 a
	D3N3	30.75 ± 0.12 a	86.68 ± 0.09 a	32.70 ± 0.04 a	5.28 ± 0.04 a	6.71 ± 0.03 a

Different lowercase letters indicate significant differences between treatments under the same fertility period according to Duncan’s test (*p* < 0.05).

## Data Availability

Data are contained within the article.
